# High Responsivity Circular Polarized Light Detectors based on Quasi Two-Dimensional Chiral Perovskite Films

**DOI:** 10.1021/acsnano.1c09521

**Published:** 2022-02-02

**Authors:** Tianjun Liu, Wenda Shi, Weidong Tang, Zilu Liu, Bob C. Schroeder, Oliver Fenwick, Matthew J. Fuchter

**Affiliations:** † School of Engineering and Material Sciences, Queen Mary University of London, Mile End Road, London E1 4NS, United Kingdom; ‡ Department of Chemistry and Molecular Sciences Research Hub, Imperial College London, White City Campus, 82 Wood Lane, London, W12 0BZ, United Kingdom; § Centre for Processable Electronics, Imperial College London, South Kensington Campus, London SW7 2AZ, United Kingdom; ∥ Department of Chemistry, University College London, 20 Gordon Street, London, WC1H 0AJ, United Kingdom; ⊥ Department of Physics, Chemistry and Biology (IFM), Linköping University, SE-581 83 Linköping, Sweden

**Keywords:** chiral perovskite, quasi-2D perovskites, photodetectors, circular dichroism, circularly polarized light

## Abstract

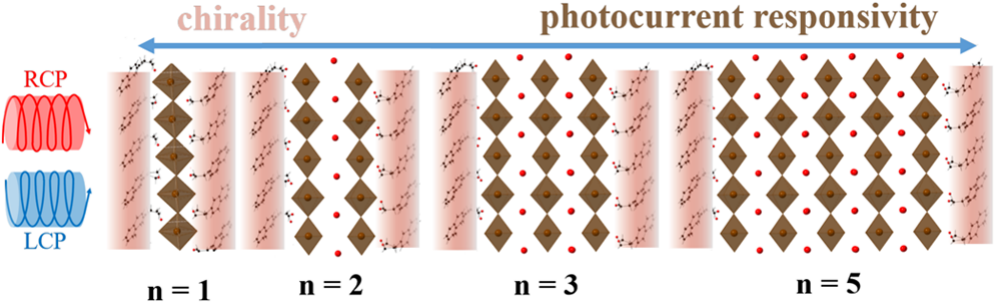

Circularly polarized light (CPL) has considerable technological potential, from quantum computing to bioimaging. To maximize the opportunity, high performance photodetectors that can directly distinguish left-handed and right-handed circularly polarized light are needed. Hybrid organic–inorganic perovskites containing chiral organic ligands are an emerging candidate for the active material in CPL photodetecting devices, but current studies suggest there to be a trade-off between the ability to differentially absorb CPL and photocurrent responsivity in chiral perovskites devices. Here, we report a CPL detector based on quasi two-dimensional (quasi-2D) chiral perovskite films. We find it is possible to generate materials where the circular dichroism (CD) is comparable in both 2D and quasi-2D films, while the responsivity of the photodetector improves for the latter. Given this, we are able to showcase a CPL photodetector that exhibits both a high dissymmetry factor of 0.15 and a high responsivity of 15.7 A W^–1^. We believe our data further advocates the potential of chiral perovskites in CPL-dependent photonic technologies.

Circularly polarized light (CPL) has attracted considerable attention due to a large number of emerging applications in many technology areas,^1^ from optical quantum computing,^2,3^ to data storage and encryption.^4^ Most commercial CPL detectors are constructed using achiral inorganic semiconductor photodetectors (which cannot detect CPL directly), in combination with a linear polarizer and quarter-wave plate. The need for additional polarization optics increases the system complexity, mechanical rigidity and difficulty in integration. As such, a high-performance CPL photodetector with the ability to distinguish the polarization states of CPL directly would provide a significant advance.

Recently, direct CPL photodetectors have been explored using chiral materials,^5^ which can distinguish left-handed circularly polarized light (LCP) and right-handed circularly polarized light (RCP). Circular dichroism (CD) measures differential absorption of LCP and RCP and therefore gives a good indication of the potential of a given material to distinguish the sign of CPL in a photodetecting device. The sensitivity of a material or device toward CPL can be assessed by the dissymmetry or “*g*” factor, which is defined as
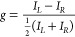
Here *L*/*R* refer to LCP and RCP illumination, and *I* can refer to a variety of measurements, for example absorbance (*g*_abs_) or responsivity (*g*_res_).

In 2010, Meskers and co-workers reported a chiral side chain polymer capable of sensing CPL in a photovoltaic device.^6^ This was followed in 2013 by Fuchter, Campbell and co-workers, who reported a CPL sensitive phototransistor employing an enantiomerically pure helicene as the active layer.^7^ The responsivity of these devices was low (<0.1 A W^–1^). There have since been several other studies concerning organic materials for photodetecting devices.^8−10^ Beyond organics, an alternative approach to a CPL detector has also been reported, which employs chiral plasmonic metamaterials.^11^ Generally, however, these devices also have low responsivity (e.g 2.2 mA W^–1^).^5^ We note that these reported responsivities are limited when considering commercial use, given that the commercial silicon photodiode CPL detector has a responsivity of around 1 A W^–1^. Therefore, it is crucial to seek advanced semiconductors with both high CP light absorption selectivity and high photoresponsivity to further enable direct CPL detection.

Hybrid organic–inorganic perovskites (HOIPs) have emerged as excellent optoelectronic semiconductors for applications in photovoltaics, light-emitting diodes, and photodetectors. HOIPs are composed of organic cations and a metal halide framework, with methylammonium lead iodide (MAPbI_3_), and formamidinium lead iodide (FAPbI_3_) being widely studied examples. After intense research of HOIP-based photovoltaics, light emitting diodes, and lasers,^12−19^ their potential for applications beyond optoelectronics, which include ferroelectrics, thermoelectrics, and spintronics, are rapidly gaining more attention.^20−26^ Accordingly, perovskite photodetectors have been reported with excellent performance compared to commercial photodetectors.^27−31^

The tunable nature of HOIPs has enabled the introduction of chiral organic ligands into the structure. In 2003, Billing and co-workers reported the synthesis of chiral organic inorganic hybrids,^32^ followed by Moon and co-workers in 2017 who systematically investigated the chiroptical properties of chiral-organic-molecule-incorporating HOIPs.^33^ Tang and co-workers then reported CPL detectors based on chiral perovskites by incorporating the chiral molecule α-phenylethylamine (α-PEA) into the HOIP structure. They obtained a device with a high responsivity of 797 mA W^–1^, but the dissymmetry factor for distinguishing RCP and LCP was low (∼0.1).^34^ More recently, Yuan and co-workers reported a flexible CPL detector using a quasi-2D structure [(*R*)-β-MPA]_2_MAPb_2_I_7_ ((*R*)-β-MPA = (*R*)-(+)-β-methylphenethylamine, MA = methylammonium) with high responsivity of 1.1 A W^–1^, and a dissymmetry factor (0.2).^35^ Alternatively, Ishii and Miyasaka reported a high dissymmetry factor (1.9) CPL detector based on a photodiode employing one-dimensional (1D) perovskite helical structure.^36^ However, the photocurrent responsivity of their device was low: 0.28 and 0.011 A W^–1^ under illumination of LCP and RCP, respectively. It therefore seems apparent that there is a trade-off between the ability to differentially absorb CPL and photocurrent responsivity in current chiral HOIP devices. Other inorganic–organic hybrid structures are also starting to be investigated in CPL detection.^37,38^

The ability to differentially absorb CPL in chiral HOIPs has been systematically studied by Sargent, Xiong, Gao, and co-workers. They used quasi-2D (also known as “Ruddlesden–Popper” structural) chiral perovskites to investigate chirality transfer as a function of the number of inorganic layers separated by chiral methylbenzylammonium organic ligands.^39^ Specifically, they studied reduced-dimensional chiral perovskites, where *n* is the average number of inorganic layers separated by bulky chiral organic ligands. The CD intensities in their work were found to inversely relate to *n*, with 200 mdeg, 20 mdeg, 3 mdeg and 1 mdeg measured for materials with *n* = 1, 2, 3, and 5, respectively. In other words, a stronger chiroptical response is observed with a lower *n* value; with the largest CD achieved for pure chiral 2D perovskites (*n* = 1) that have the highest mole fraction of chiral organic ligands. Conversely, in the quasi-2D perovskite solar cell devices, a high *n* value results in a high photocurrent.^40−42^ This data once again supports a trade-off between the ability to differentially absorb CPL and photocurrent responsivity in current chiral HOIP devices. It remains unclear as to whether it is possible to overcome this apparent dichotomy and achieve chiral HOIP CPL detectors with both high dissymmetry and good photocurrent responsivity.^43^

Here, we report the synthesis of quasi-2D chiral HOIPs using the chiral molecule 1-(2-naphthyl)ethylamine (NEA, Figure 1a), which has previously shown promise in chiral HOIP materials with tunable CD.^44^ A structural isomer of the chiral ligand we use in this study was also recently reported in HOIP devices that are able to emit room temperature circular polarized photoluminescence.^45^ In contrast to this prior work, we have investigated our quasi-2D perovskite (NEA)_2_(MA)_n-1_Pb_n_I_3n+1_ films for CPL detection in devices. Strikingly and despite the prior art, we find that there is the potential to fine-tune the *n* value in our material in order to achieve good dissymmetry and photocurrent responsivity simultaneously. Specifically, our chiral HOIP materials result in the high CPL dissymmetry and photoresponsivity, achieving maximum dissymmetry factor of responsivity (*g*_res_) of 0.15 together with high responsivity of 15.7 A W^–1^. We believe our work further advocates the development of chiral HOIP materials in high performance direct CPL detectors.

**Figure 1:**
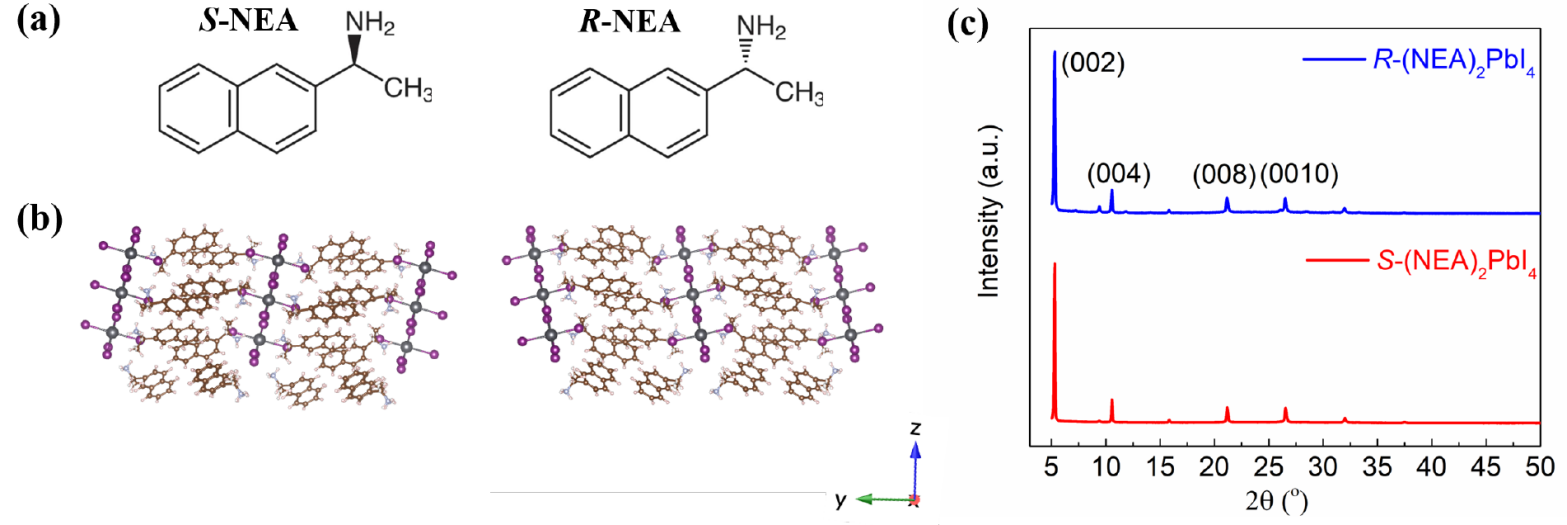
Structural data: (a) structures of the chiral organic molecules used in this work. (b) the crystal structure of (*S*-NEA)_2_PbI_4_ (left) and (*R*-NEA)_2_PbI_4_ (right) viewed along the *x*-axis. (c) the XRD patterns of (*R-/S-*NEA)_2_PbI_4_ films on quartz.

## Results and Discussion

2D (*S*-/*R*-NEA)_2_PbI_4_ (*n* = 1) films were fabricated on quartz substrates by spin coating the precursors and annealing in nitrogen atmosphere. The difference between the *S*- and *R*-structures (Figure 1a) comes from incorporating the *S* and *R* enantiomers of NEA, respectively. The crystal structure of (NEA)_2_PbI_4_ HOIP is shown in Figure 1b. The [PbI_6_]^4–^ octahedra share four corners at the halide position and form inorganic layers in the *xy* plane. The chiral organic cation, NEA, forms the organic layers in a similar manner to the reported 2D perovskites based on phenylethylammonium (PEA) and methylbenzylammonium (MBA).^46,47^ Due to the large π-conjugated naphthalene skeleton in NEAI, the neighboring molecules in the crystal structure have strong interactions and thus strongly affect the helicity of [PbI_6_]^4–^ octahedral cages.^47^ X-ray diffraction (XRD) of *S*- and *R*-(NEA)_2_PbI_4_ (*n* = 1) films show clear peaks at 5.2°, 10.5°, 21.2°, and 26.4°, which correspond to the (002), (004), (008), and (0010) planes, respectively, indicating a preferential crystallite orientation with the *c* axis perpendicular to the substrate (Figure 1c). The full width at half-maximum of the (002) peak is 0.13° and 0.09° for *S*- and *R*-(NEA)_2_PbI_4_ films, respectively, indicating high crystallinity. Based on these *n* = 1 films, we further investigated quasi-2D perovskite films by introducing methylammonium cations MA^+^ into the film structure: (*S*-/*R*-NEA)_2_(MA)_n-1_Pb_n_I_3n+1_ structure (*n* = 2, 3, 5). (NEA)_2_(MA)Pb_2_I_7_ (*n* = 2), (NEA)_2_(MA)_2_Pb_3_I_10_ (*n* = 3) and (NEA)_2_(MA)_4_Pb_5_I_16_ (*n* = 5) were produced in a similar manner to (NEA)_2_PbI_4_ (*n* = 1), but from solutions containing an appropriate stoichiometry of (NEA)I, PbI_2_ and methylammonium iodide (MAI) (see Supporting Information (SI)). XRD spectra (SI) show the main peak of the 2D perovskite structure at 5.2° in both fresh films and films aged in air without encapsulation, which means the 1S film shows a good stability over a period of one month. XRD spectra of the 3*S* films indicate a similar stability over a period of one-month.

**Figure 2:**
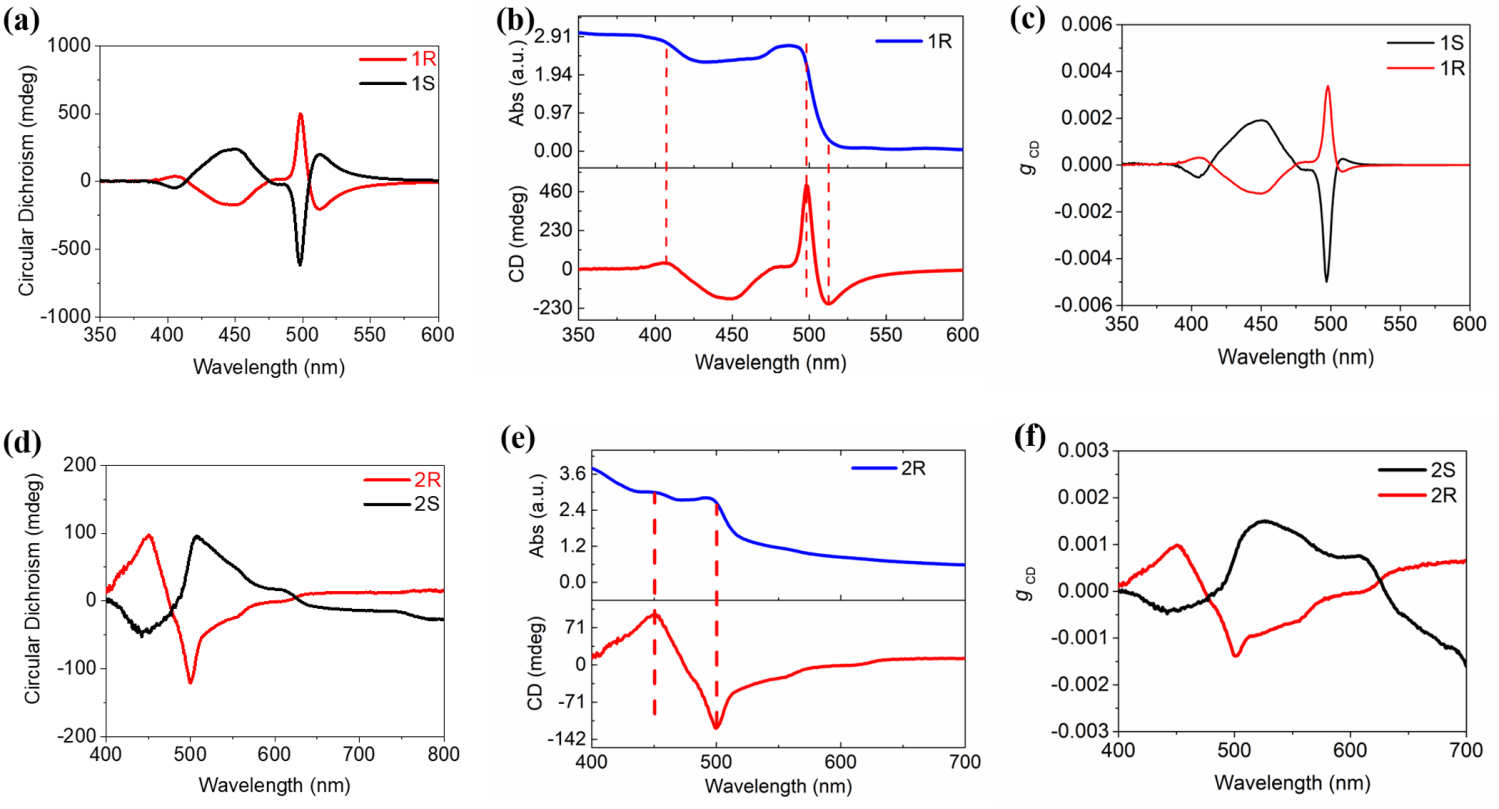
Characterization of chiral optical properties of the (*R*-*S*-NEA)_2_(MA)_*n*−1_Pb_*n*_I_3*n*+1_ perovskite films. (a) and (d) CD of the films with *n* = 1 and 2, respectively. (b) and (e) UV–vis absorption spectra of 1*R* and 2*R*, respectively. (e) and (f) the dissymmetry factor *g*_CD_ of the films with *n* = 1 and 2.

The UV–vis spectra of the (*S*-NEA)_2_(MA)_n-1_Pb_n_I_3n+1_ perovskite films with *n* = 1, 2, 3, and 5 are shown in the SI. For *n* = 1, the absorption peak is broadened at 476 nm and the absorption edge is 505 nm, which is consistent with previously reported work.^44^ For *n* = 2, 3, and 5 films, the spectra show typical multiple exciton absorption peaks of quasi-2D perovskites.^41,42^ To characterize the chiroptical properties of our materials, we performed CD measurements as shown in Figure 2a–f. The CD peaks of samples containing an *S* and *R* configuration of the organic ligand, at corresponding wavelengths, are of opposite sign. For the *n* = 1 sample in Figure 2a, the CD spectra show a peak at 450 nm with an intensity of −178 mdeg and 244 mdeg for *R* and *S*, respectively. Variations in the magnitude of the CD response for *S* and *R* organic ligands are consistent with previous work.^44^ The CD intensity in chiral perovskites is 2 orders of magnitude larger than that of chiral ligands reported in a previous study,^44^ indicating the chirality has been successfully transferred from organic ligands to HOIPs. When *n* is increased to 2 via the introduction of MA^+^ cations to form the quasi-2D structure, the CD spectra show a peak at 450 nm with an intensity of 98 mdeg and −52 mdeg for *R* and *S*, respectively in Figure 2d. The CD spectra in the *n* = 3 films show a weaker peak of 40 mdeg at 390 nm and for *n* = 5, the CD signal is less than 5 mdeg (SI). This trend to lower CD intensities for larger *n* is consistent with reported work.^39^ The dissymmetry of absorption (*g*_abs_) or (*g*_CD_) can be calculated by the following equation:
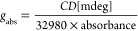


The *g*_abs_ values of our films indicate that the induced chiroptical activity in the HOIPs is approximately the same magnitude in *n* = 1 and 2 samples, as shown in Figure 2c,f. Given the chiroptical response of our materials, particularly as a function of *n*, we proceeded to investigate their use in devices.

**Figure 3:**
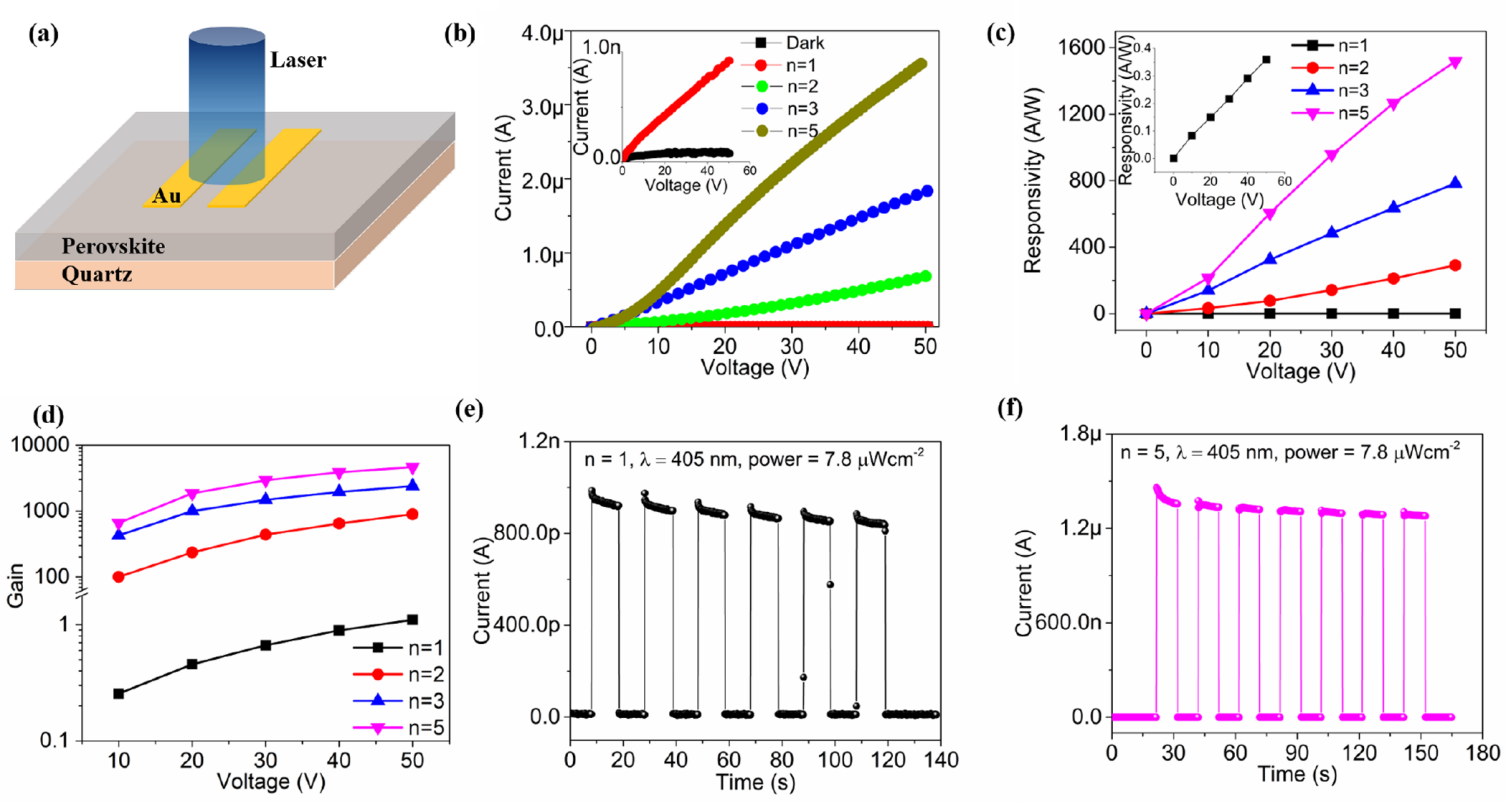
Photodetector characterization of devices constructed from (*S*-NEA)_2_(MA)_*n*−1_Pb_*n*_I_3*n*+1_ perovskite films. (a) schematic diagram of photodetector device based on planar structure with gold contacts. (b) *I–V* curves of the device in dark and below a 405 nm illumination (irradiance 7.8 μW cm^–2^) of various perovskite structures with *n* = 1, 2, 3, and 5. Insert plot is the *I–V* curve of the device with perovskite structure of *n* = 1. (c) responsivity and (d) photocurrent gain as a function of the bias voltage from 0 to 50 V of perovskite structures with *n* = 1, 2, 3, and 5. Insert plot in (c) is the responsivity of the device with perovskite structure of *n* = 1. (e) and (f) time-resolved response of the device in darkness and under light illumination at a 20 V bias with *n* = 1 and *n* = 5, respectively.

We investigated device performance based on our chiral perovskites under unpolarized light. Photodetector devices were fabricated using our quasi-2D (*S*-NEA)_2_(MA)_*n*−1_Pb_*n*_I_3*n*+1_ perovskite films, with gold (50 nm) as the source and drain electrodes (Figure 3a). The current–voltage (I–V) curves of the devices, in the dark and under 405 nm illumination, show the photocurrent to be proportional to the *n* value and the drain voltage (Figure 3b), consistent with a previous study.^48^ We extracted photoresponsivity (R), which is a significant photodetector parameter by using the equation:
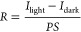
Where *I*_light_ and *I*_dark_ are the current under illumination and in the dark, respectively. *P* is the incident power density, *S* is the effective area being illuminated. For *n* = 1, the responsivity shows a high value of 0.15 A W^–1^ at a bias of 20 V with photocurrent gain of 0.46 (Figure 3c). For quasi-2D structures, the responsivity shows an increasing trend with *n* value from 2 to 5, achieving a high responsivity (*n* = 5) of 606 A W^–1^ at bias of 20 V with photocurrent gain of 1860. The highest responsivity is achieved of 1520 A W^–1^ at bias of 50 V for *n* = 5. We are only aware of one previous study where quasi-2D chiral HOIP structures were used in devices (*R*-MPA_2_MAPb_2_I_7_, *R*-MPA = *R*-methylphenethylamine),^34^ which measured peak responsivities of 3.8 A W^–1^ (*n* = 2) at 10 V and 0.797 A W^–1^ (*n* = 1) at 10 V; significantly lower than the values measured for our materials. We find that the photocurrent gain increases with bias voltage and further increases as a function of the *n* value (Figure 3d). This suggests that the photocurrent could be enhanced by increasing the 3D phase composition in quasi-2D perovskite structures. A stable response to light is observed, with identical current levels observed for several cycles in the time-dependent experiments (Figure 3e,f). The dark current in the *n* = 1 perovskite structure is 1.2 × 10^–11^ A at 20 V, whereas in the perovskite structure with *n* = 3 it is 1 order of magnitude higher at 1.6 × 10^–10^ A at 20 V. The low dark currents in these materials are likely due to the low density of intrinsic free charge carriers in layered perovskites^35^ which is lowest for *n* = 1. Overall, this indicates excellent stability, reversibility, and photosensitivity of our quasi-2D chiral perovskite photodetectors.

**Figure 4:**
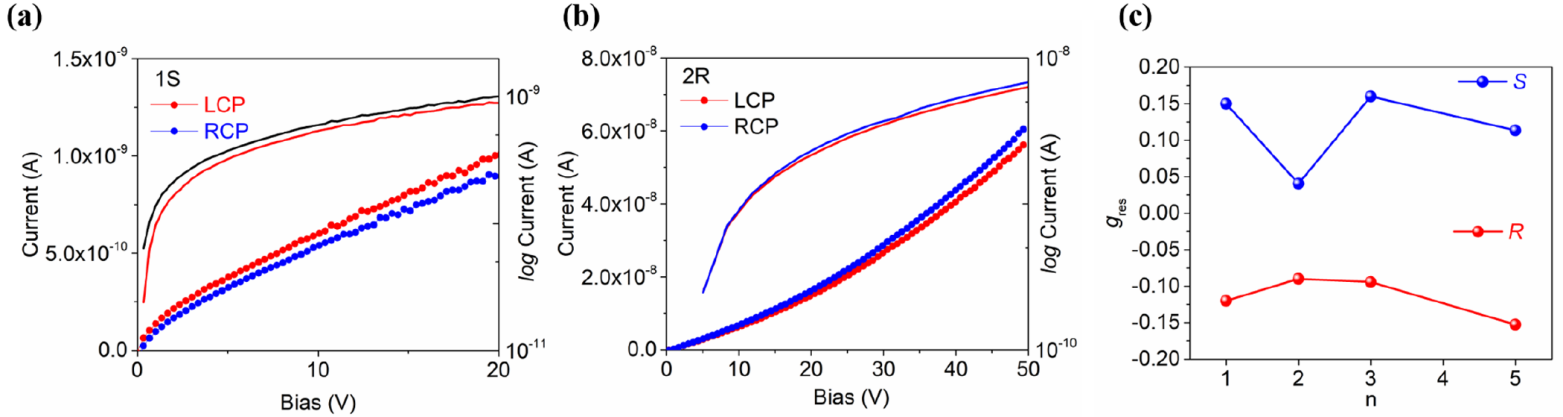
Performance of (*S*- and *R*-NEA)_2_(MA)_n-1_Pb_*n*_I_3*n*+1_ perovskite CPL photodetectors. (a) and (b) the *I–V* curve of (*S-R-*NEA)_2_(MA)_*n*−1_Pb_*n*_I_3*n*+1_ perovskite device under dark, LCP-405 and RCP-405 nm light illumination with 1*S* and 2*R*, respectively. The light intensity was 7.8 μW cm^–2^. (c) *g*_res_ as a function of *n* in (*R*-NEA)_2_(MA)_*n*−1_Pb_*n*_I_3*n*+1_ (red) and *(S*-NEA)_2_(MA)_*n*−-1_Pb_n_I_3*n*+1_ (blue) perovskite.

To investigate CPL detection, we used instrumentation reported in our previous work.^10^ When using the *S* enantiomer of NEA in our HOIPs, the photocurrent generated by LCP illumination at 405 nm is larger than when using RCP illumination (Figure 4a), indicating the different responsivity to RCP and LCP photons. When using the *R* enantiomer of NEA in perovskites films, the photocurrent generated by RCP is larger than the one generated by LCP for *n* = 3 structures (Figure 4b). To quantify CPL detection in our photodetectors, the dissymmetry factor of responsivity, *g*_res_, was used.^34^ We investigated the *g*_res_ at bias range from 0 to 50 V with 1*S* devices. As shown in the I–V curve (SI), in low bias range from 0 to 12 V, the I−V response is due to traps rather than CPL generated carriers (SI). At a higher bias range (12 to 50 V), we assume that the I−V curve represents the transport of charge carriers induced by CPL. Thus, we extract the *g*_res_ at a high bias of 40 V. As shown in Figure 4c, *g*_res_ maintains the same magnitude as *n* increases from 1 to 5, where the *n* = 3 samples exhibit high responsivity of 15.7 A W^–1^ at 2.8 V. Our device performance is comparable to state-of-art CPL photodetectors reported to date, as shown in Table 1. We also found the sign of *g*_res_ to invert when the opposite enantiomer of NEA was used, as would be expected. Meanwhile, we investigated the device stability of the 1*S* sample as shown in the SI. The current generated from CPL has a slightly decrease of 10% in aged samples kept with room temperature in air) compared to fresh ones.

**Table 1:** A Summary of CPL Detectors Based on Chiral Perovskites

CPL detector	responsivity (A W^–1^)	*g*_CD_	*g*_res_ or *g*_I_	stability	ref
(*S-*/*R*-PEA)PbI_3_	0.797	0.02	0.1	one month	(34)
(*S-*/*R-*1–1-NEA)PbI_3_	0.28	0.04	1.8		(36)
(*S-*/*R-*MPA)_2_MAPb_2_I_7_	1.1		0.2		(35)
(*S-*/*R-*BPEA) _2_PbI_4_	0.002	0.003	0.13		(49)
(*S-*/*R*-PEA)_2_PbI_4_	0.6	N/A	0.23		(50)
(*S-*/*R-*1–2-NEA) MAPb_2_I_7_	15.7	0.005	0.15	one month	this work

**Figure 5:**
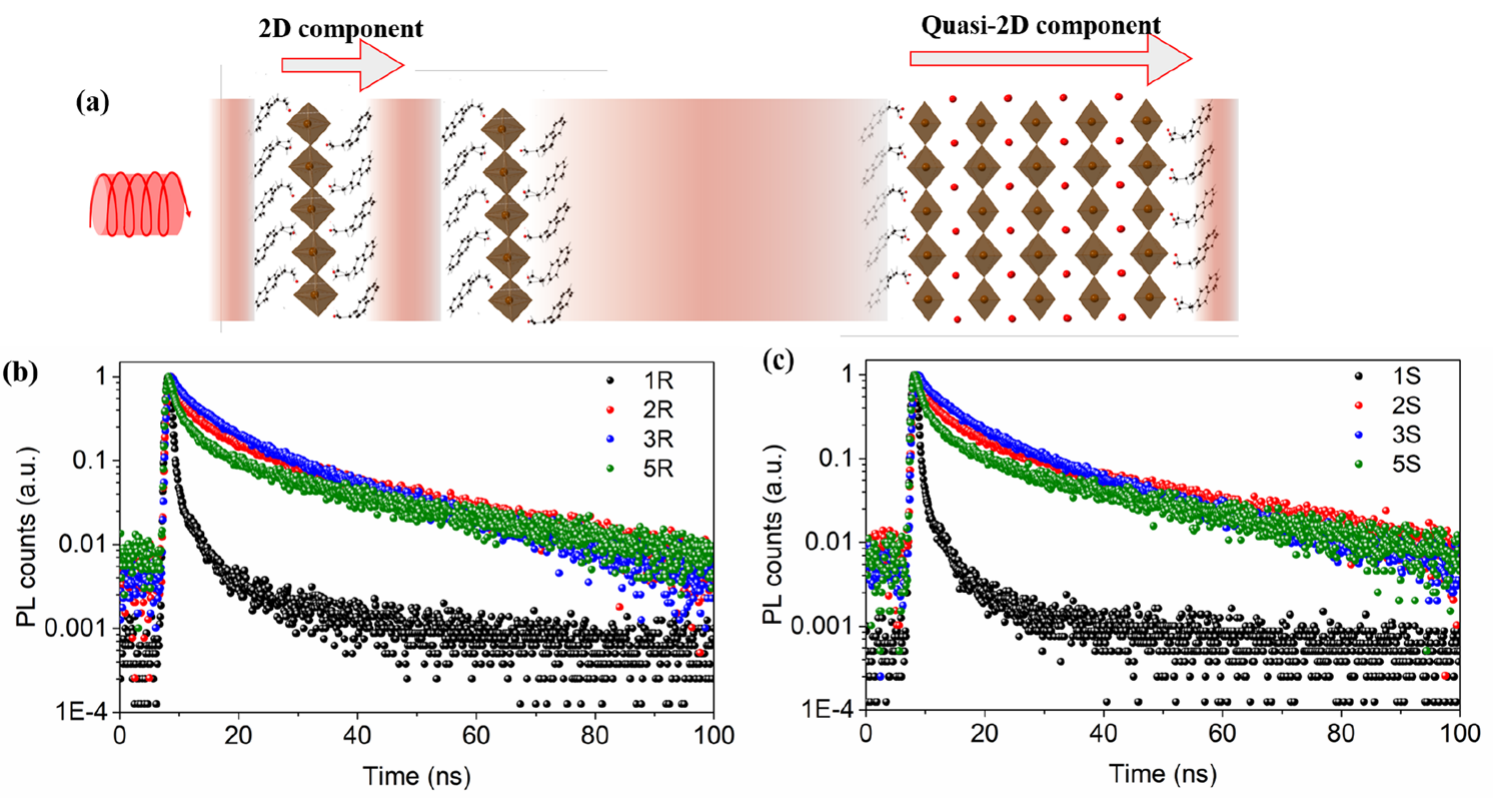
Circularly polarized light induced carrier kinetics in 2D and quasi-2D chiral perovskite films. (a) Schematic of CPL-induced carrier transport in 2D and quasi-2D components. (b) and (c) time-correlated single-photon counting spectra of (*R*-NEA)_2_(MA)_*n*−-1_Pb_n_I_3*n*+1_ and (*S*-NEA)_2_(MA)_*n*−1_Pb_n_I_3*n*+1_, respectively. *n* = 1, 2, 3 and 5.

The trend in the dissymmetry factor of CPL detection by our devices (*g*_res_) is different to the trend of the dissymmetry factor of absorption (*g*_CD_). Generally speaking, this outcome is not uncommon in prior examples of CPL detecting devices^36^ and further work is needed to fully elucidate the mechanisms at play. Nonetheless, we believe that the interplay between the number of photoinjected charge carriers and the charge transport of the material plays a key role here. To study this aspect further, we investigated the lifetime of the charge carriers in both 2D and quasi 2D films by using time correlated single photon counting (TCSPC). As shown in Figure 5, the 2D films show a short PL lifetime of 4.0 ns, while the lifetimes of 2*R*, 3*R*, 4*R*, and 5*R* films are 19.1 ns, 16.1 ns, 17.9 ns, and 17.2 ns, respectively. It is therefore clear that the photogenerated carriers have larger lifetime in quasi-2D films. Due to strong electron–phonon coupling effects in 2D perovskite structures,^18^ the carrier lifetime in these structures is usually short. Quasi-2D perovskites on the other hand have weaker electron–phonon coupling and can undergo fast transfer of the carriers generated to the high *n* value component, MAPbI_3_, in an energy funneling process. We believe such energy funneling may contribute to the amplified *g*_res_ of our quasi-2D devices.

## Conclusions

In conclusion, we have successfully demonstrated a direct CPL detector based on quasi-2D chiral perovskites (*R*-, *S*-NEA)_2_(MA)_*n*−1_Pb_*n*_I_3*n*+1_ films. In contrast to prior studies, our HOIP films exhibit comparable CD intensity for *n* = 1 to 3 (from 2D to quasi-2D structures), which suggests it may be possible to obtain high responsivity and high dissymmetry simultaneously from such materials. Indeed, we showcase a quasi-2D HOIP CPL photodetectors with a maximum dissymmetry factor of responsivity (*g*_res_) of 0.15 together with high responsivity of 15.7 A W^–1^. We believe our work should further boost the recent interest in direct CPL detection based on quasi-2D perovskites and other chiral optoelectronic devices based on chiral perovskites.

## Experimental Section

### Materials

PbI_2_ (99.99%) and chiral (*S*, *R*)1-(2-naphthyl)ethylamine (NEA) were purchased from TCI. Methylammonium iodide (MAI) (>99%) was purchased from Greatcell Solar. DMF, DMSO and chlorobenzene were purchased from Sigma-Aldrich. Hydroiodic acid, methanol, ethanol and other solvents were purchased from Fisher Scientific. All chemicals were directly used without further purification. Quartz substrates were obtained from Ossila.

### Chiral NEAI Synthesis

(*S*, *R*) 1-(2-naphthyl)ethylamine (3 mmol, 0.513g) and MeOH (10 mL) were added to a 500 mL flask. The solution was cooled to 0 °C and put under an argon atmosphere by gas flow for 10 min. HI (1 mL, 4.5 mmol, 1.5 equiv) was then added dropwise. The solution was stirred for 2 h. Concentration of the reaction mixture gave a white solid. Diethyl ether was added and the suspension was stirred for 15 min. The solid was filtered and was washed with diethyl ether to provide (*S*, *R*) 1-(2-naphthyl)ethylammonium iodide (*S*-, *R*-NEAI) as a white solid.

### Thin Film Deposition

Perovskite thin films were prepared by spin coating the precursor solution at 3000 rpm for 30 s followed by dripping 200 μL of chlorobenzene as an antisolvent onto the sample. The films were then annealed at 100 °C for 30 min for crystallization residual solvent removal. The thickness of our 1*S* and 1*R* perovskite films is 280 nm. The thickness of quasi-2D films are 340–400 nm.

### Device Fabrication

The substrates were cleaned with deionized water, acetone and isoproposal for 15 min each. The precleaned substrates were placed in a UV-zone cleaner for 30 min before spin coating. The active layer was deposited by spin coating the precursor solution as the method mentioned above. 80 nm thick gold was thermally evaporated as top contacts in a two terminal planar electrode structure with channel length of 30 μm and width of 1 mm.

### CPL Photodetector Measurement Setup

CPL was generated using a wire grid linear polarizer (WP25M-VIS, Thorlabs) and a 405 nm quarter-wave plate (WPMQ05M-405, Thorlabs), which has been reported in our previous work.^10^ All the measurements were performed in a glovebox.

### Photoluminescence Measurements

Time-correlated single photon counting (TCSPC) measurements were carried out by using an Edinburgh Instruments FL1000 with a 405 nm pulsed picosecond laser (EPL-405). Steady state PL was performed with this Edinburgh Instruments FL1000 by recording excitation–emission data.

## Supporting Information Available

Additional CD and CPL photodetector data (PDF)
