# Supplementary material for: High Responsivity Circular Polarized Light Detectors based on Quasi Two-Dimensional Chiral Perovskite Films
Source: ACS Nano. 2022 Feb 2;16(2):2682–9. doi: 10.1021/acsnano.1c09521 (PMC9007523; doi:10.1021/acsnano.1c09521)
Supplement: Supplementary file 1 — nn1c09521_si_001.pdf [file nn1c09521_si_001.pdf]

Supplementary Information for

High Responsivity Circular Polarized Light Detectors based on Quasi Two-Dimensional Chiral Perovskite Films

Tianjun Liu[1,5#], Wenda Shi[2#], Weidong Tang[1], Zilu Liu[4], Bob C. Schroeder[4], Oliver Fenwick[1], and Matthew J. Fuchter[2,3*]*

Perovskite precursor solutions. 1M NEAI solution was prepared by adding 1 mmol NEAI in 1mL DMF. 1.5 M MAI solution was prepared by adding 1.5 mmol MAI in 1 mL DMF. 1.5 M PbI_2 solution was prepared by adding 1.5 mmol PbI_2 in 1 mL DMF. All solutions were prepared in the glovebox with only DMF solvent.

For $n = 1$ sample, $(NEA)_2PbI_4$, 250 μL NEAI, 84 μL PbI_2 and 166 μL DMF were mixed together.

For $n = 2$ sample, $(NEA)_2(MA)Pb_2I_7$, 250 μL NEAI, 84 μL MAI, 167 μL PbI_2 were mixed together.

For $n = 3$ sample, $(NEA)_2(MA)_2Pb_3I_{10}$, 162 μL NEAI, 111 μL MAI, 167 μL PbI_2 and 55 μL DMF were mixed together .

For $n = 5$ sample, $(NEA)_2(MA)_4Pb_5I_{16}$, 100 μL NEAI, 133 μL MAI, 167 μL PbI_2 and 100 μL DMF were mixed together .

Structure properties of quasi-2D perovskite thin films.

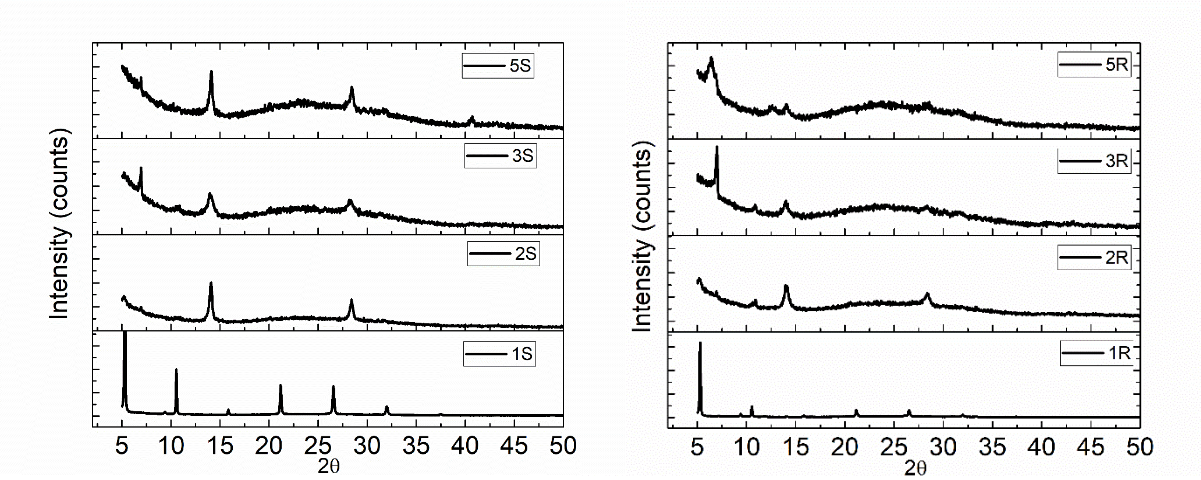

Figure S1. XRD spectra of quasi-2D chiral perovskites, $(NEA)_2(MA)_{n-1}Pb_nI_{3n+1}$, n =1,2, 3 and 5.

Chiral optical properties of quasi-2D perovskite thin films.

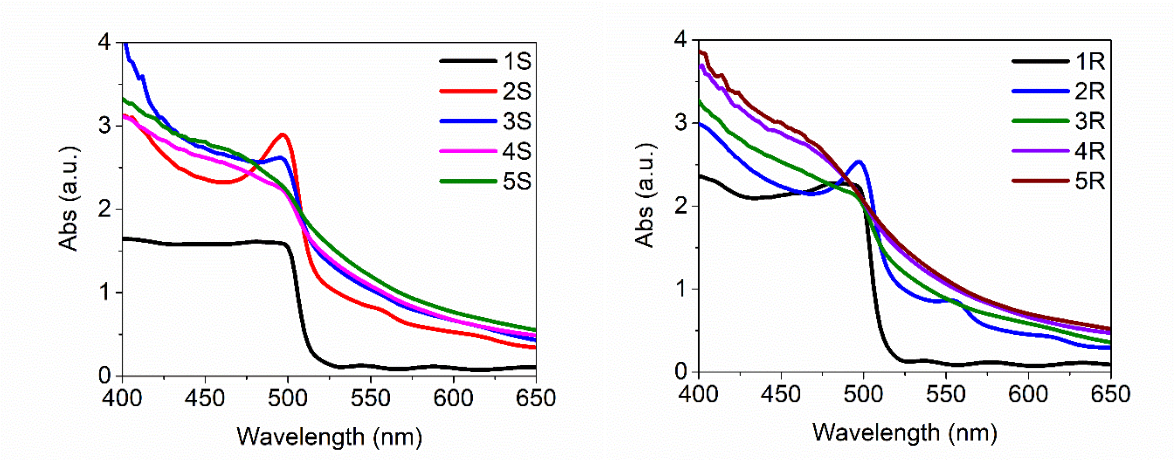

Figure S2. UV-vis absorption spectra of (*S-/R*-NEA)$_2$(MA)$_{n-1}$Pb$_n$I$_{3n+1}$, n =1,2, 3, 4 and 5.

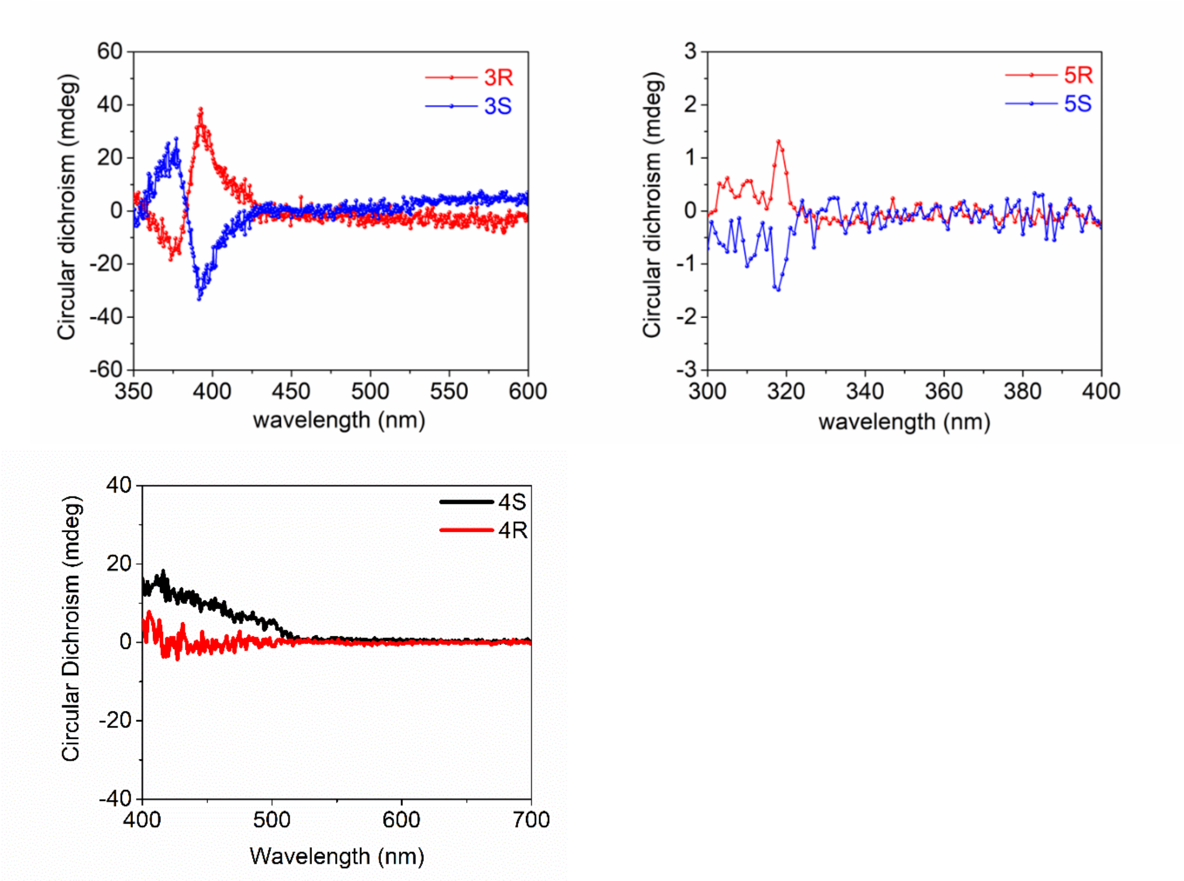

Figure S3. CD of the films with n = 3,4 and 5.

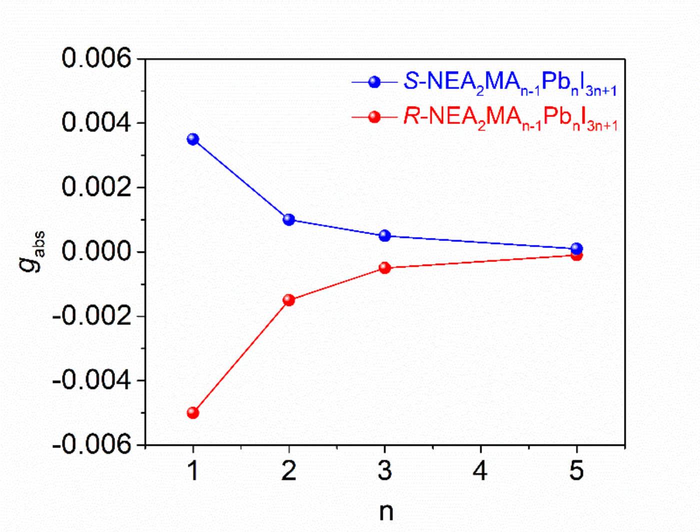

Figure S4. g_{CD} as the value of n from 1 to 5 at the local maximum point.

Photoluminescence of quasi-2D films.

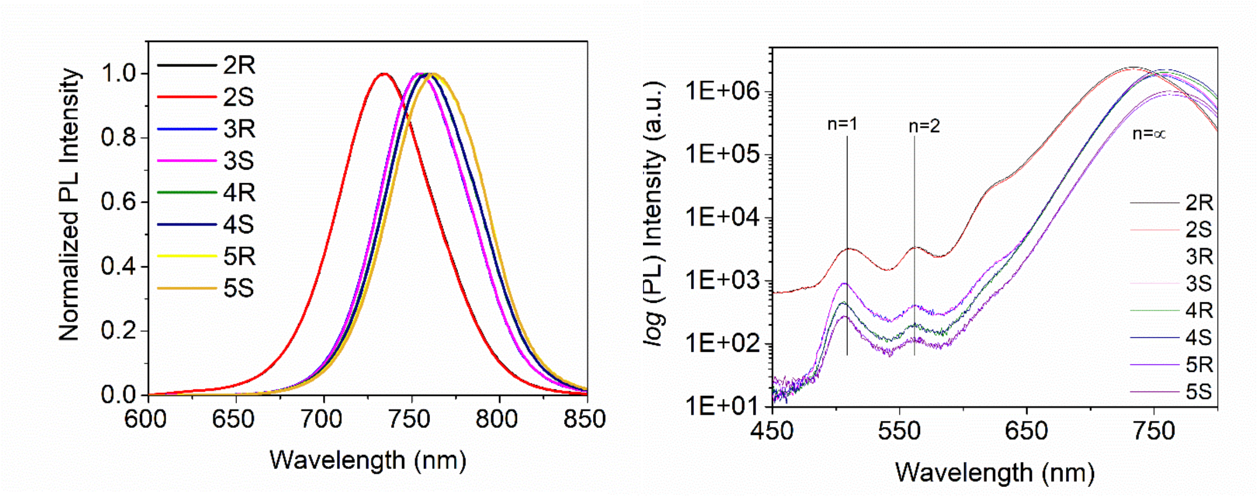

Figure S5. PL spectra of perovskites $(NEA)_2(MA)_{n-1}Pb_nI_{3n+1}$, n =2, 3, 4 and 5.

Phase purity of quasi-2D chiral perovskite films.

To investigate the phase purity in our samples, we have employed excitation-emission spectroscopy for our samples. In front illumination, the incident light goes through the film towards the substrate, whereas in back illumination mode, light goes through the substrate to the film. In our setup, we use excitation from 360 nm to 600 nm. In this range, all the phases of the quasi-2D structure can be excited and give the related emission data in our spectra. Vertical phase segregation would result in different excitation-emission spectra for front and back illumination. Here, we show data for the chiral samples with n=3 and 5 of S- chiral perovskite films. As shown below, the emission map of a 3S film measured in front and back modes has a single emission at 760 nm. For 5S samples, the excitation-emission spectrum is also similar under front and back illumination mode with single emission at 770nm. Multiple emission peaks have not been observed in our quasi-2D films, and similarity between spectra in front- and back-illumination geometry suggests that there is no vertical phase segregation.

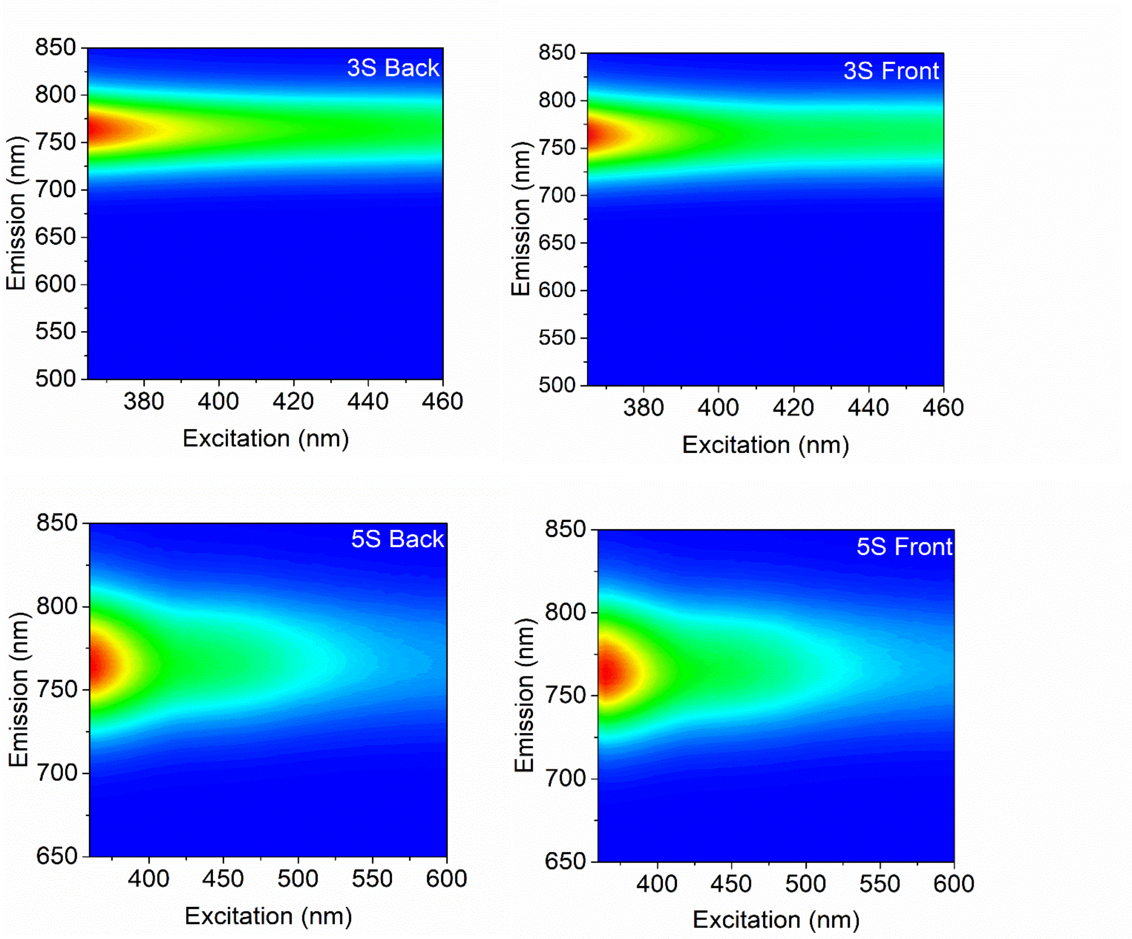

Figure S6. Excitation-emission map of quasi-2D chiral perovskite films with n = 2 and n = 5.

CPL photodetector performance.

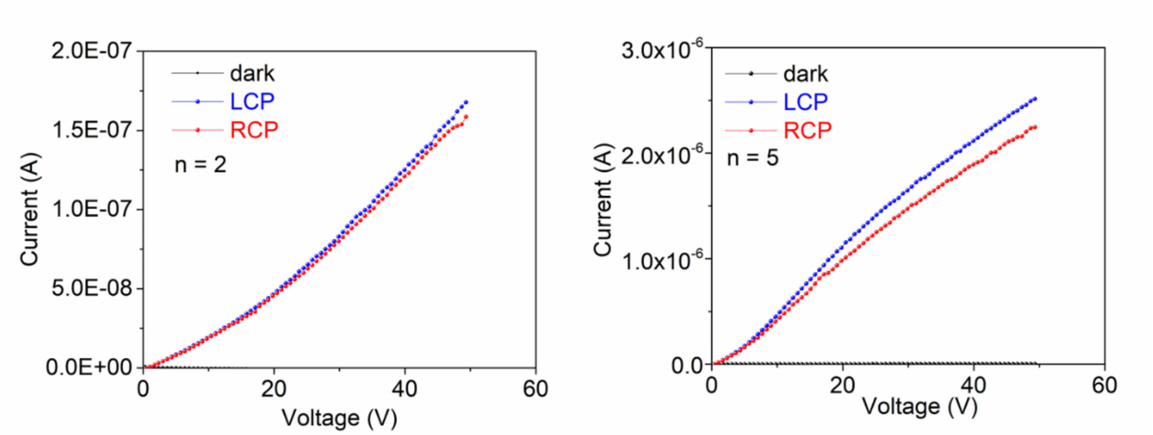

Figure S7. *I-V* curve of quasi-2D perovskite device under dark and LCP 405 nm and RCP 405 nm light illumination with n = 2 and n = 5, respectively. The light intensity was 7.8 μW cm^{-2}.

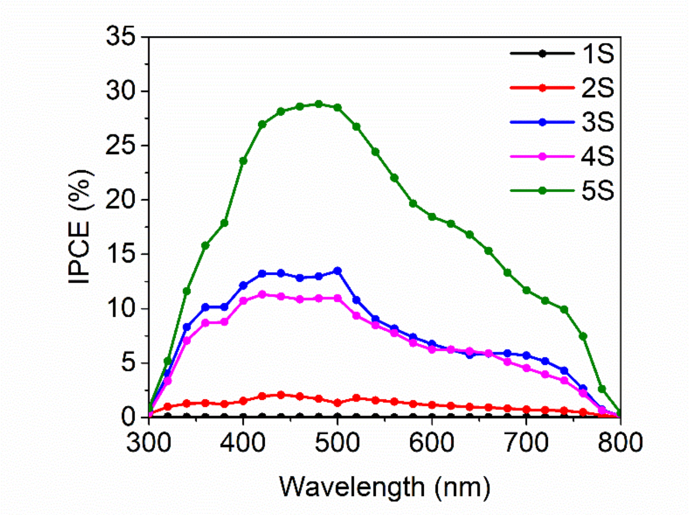

Figure S8. The incident photon to current conversion efficiency (IPCE) of $(S\text{-}NEA)_2(MA)_{n-1}Pb_nI_{3n+1}$ from n=1 to 5.

Photocurrent dissymmetry factor as a function of bias voltage dependence.

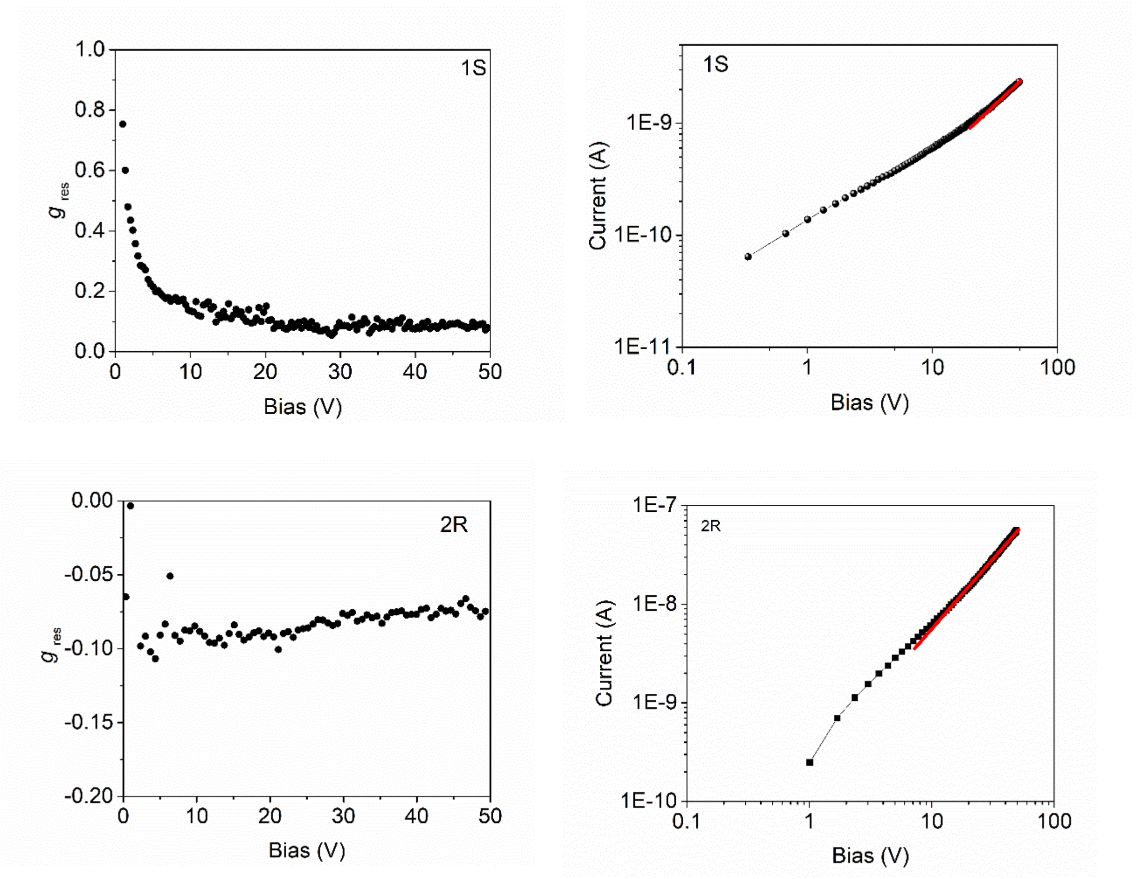

Figure S9. Photocurrent dissymmetry factor under low and high bias range.

Stability measurement of film and CPL detectors.

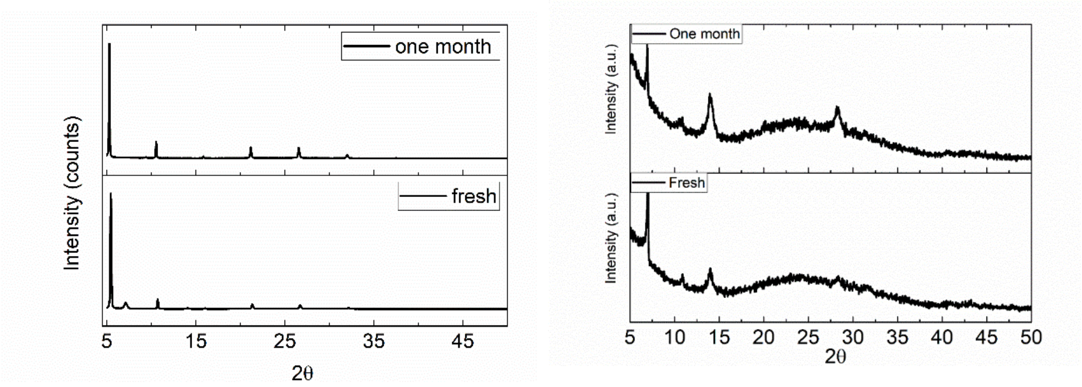

Figure S10. XRD measurement of 1S films (left) and 3S films (right).

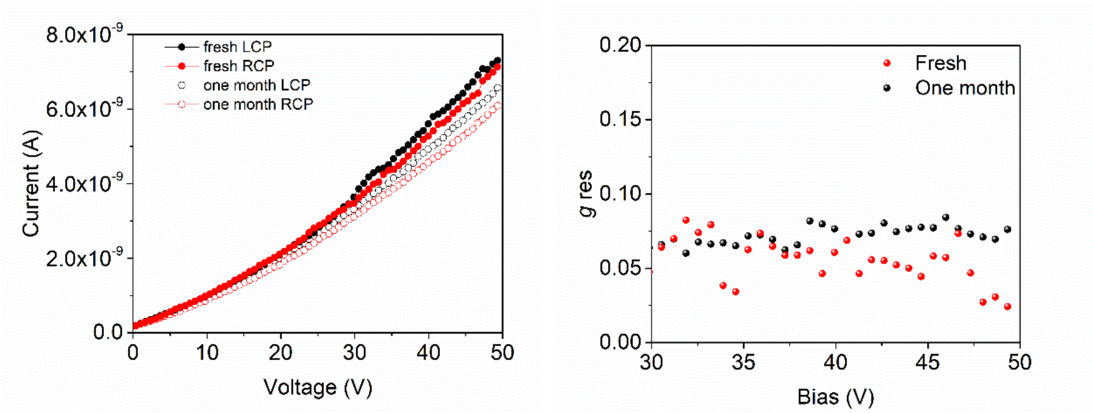

Figure S11. Stability of CPL detector based on 1S sample. I-V curve (left) and g factor-V curve. (right)

REFERENCE

1. Shi, W.; Salerno, F.; Ward, M. D.; Santana-Bonilla, A.; Wade, J.; Hou, X.; Liu, T.; Dennis, T. J. S.; Campbell, A. J.; Jelfs, K. E.; Fuchter, M. J. Fullerene Desymmetrization as a Means to Achieve Single-Enantiomer Electron Acceptors with Maximized Chiroptical Responsiveness. *Adv. Mater.* **2021**, *33* (1), 2004115. https://doi.org/10.1002/adma.202004115
